# Plasma Free Hemoglobin Is an Independent Predictor of Mortality among Patients on Extracorporeal Membrane Oxygenation Support

**DOI:** 10.1371/journal.pone.0124034

**Published:** 2015-04-22

**Authors:** Hesham R. Omar, Mehdi Mirsaeidi, Stephanie Socias, Collin Sprenker, Christiano Caldeira, Enrico M. Camporesi, Devanand Mangar

**Affiliations:** 1 Department of Internal Medicine, Mercy Medical Center, Clinton, Iowa, United States of America; 2 Division of Pulmonary, Critical Care, Sleep and Allergy, Department of Medicine, University of Illinois at Chicago, Chicago, IL, United States of America; 3 Department of Research, Florida Gulf-to-Bay Anesthesiology Associates, Tampa, Florida, United States of America; 4 Department of Cardiothoracic Surgery, Florida Advanced Cardiothoracic Surgery, Tampa, Florida, United States of America; 5 University of South Florida, FGTBA and TEAMHealth, Tampa, Florida, United States of America; 6 Department of Anesthesia, Tampa General Hospital, FGTBA, TEAMHealth, Tampa, Florida, United States of America; Sapienza University of Rome, ITALY

## Abstract

**Background:**

Hemolysis is common in all extracorporeal circuits as evident by the elevated plasma free hemoglobin (PFHb) level. We investigated whether increased hemolysis during extracorporeal membrane oxygenation (ECMO) is an independent mortality predictor.

**Methods:**

We performed a retrospective observational study of consecutive subjects who received ECMO at a tertiary care facility from 2007-2013 to investigate independent predictors of in-hospital mortality. We examined variables related to patient demographics, comorbidities, markers of hemolysis, ECMO characteristics, transfusion requirements, and complications. 24-hour PFHb> 50 mg/dL was used as a marker of severe hemolysis.

**Results:**

154 patients received ECMO for cardiac (n= 115) or pulmonary (n=39) indications. Patients’ mean age was 51 years and 75.3% were males. Compared to nonsurvivors, survivors had lower pre-ECMO lactic acid (p=0.026), lower 24-hour lactic acid (p=0.023), shorter ECMO duration (P=0.01), fewer RBC transfusions on ECMO (p=0.008) and lower level of PFHb 24-hours post ECMO implantation (p=0.029). 24-hour PFHb> 50 mg/dL occurred in 3.9 % versus 15.5% of survivors and nonsurvivors, respectively, p=0.002. A Cox proportional hazard analysis identified PFHb> 50 mg/dL 24-hours post ECMO as an independent predictor of mortality (OR= 3.4, 95% confidence interval: 1.3 – 8.8, p= 0.011).

**Conclusion:**

PFHb> 50 mg/dL checked 24-hour post ECMO implantation is a useful tool to predict mortality. We propose the routine checking of PFHb 24-hours after ECMO initiation for early identification and treatment of the cause of hemolysis.

## Introduction

Extracorporeal membrane oxygenation (ECMO) has shown promising results for critically ill patients, requiring cardiopulmonary support, who are otherwise expected to die [1,2]. Patients receiving ECMO support experience an approximate 60–75% in-hospital mortality rate [3–6] due to severity of the underlying pathology and high incidence of multiple organ failure, despite improvement in intensive care management and advances in ECMO hardware technology. The availability of new treatment modalities for refractory circulatory failure including ventricular assist device and cardiac transplantation expand the role of ECMO as a bridge to these procedures. The high cost and low survival rates in ECMO subjects mandate an awareness of determinants of adverse outcomes for ideal patient selection to avoid providing futile care.

Although mortality from ECMO is mainly determined by ECMO-unrelated factors (e.g severity of main underlying pathology, number of affected organs), it is also affected by ECMO-related complications such as hemorrhagic events [7], the need for renal replacement therapy [8] during ECMO support, venoarterial ECMO mode [9] and the amount of blood transfused during ECMO support [9]. Hemolysis is common in extracorporeal circuits as evident by the elevated plasma free hemoglobin (PFHb) level [10,11]. Hemolysis in ECMO circuits occur due to a variety of reasons including the negative pressure generated by the pump in hypovolemic states, the development of a clot within the circuit or near the cannula orifices or excessive centrifugal pump speed >3000 revolutions per minute (RPM) [12].

Adverse outcomes related to high levels of PFHb can be explained by its propensity to cause direct kidney injury because of renal tubular obstruction, the need for more blood transfusion with the risk of transfusion-related complications and even death in children requiring ECMO after cardiac surgery [13]. There are also concerns of a possible association between high PFHb level and multiple organ failure [14,15]. No study has examined the relationship between PFHb and survival in adults receiving ECMO support. The main objective of this study is to determine mortality predictors in subjects receiving ECMO for various cardiac or pulmonary indications and whether increased hemolysis in ECMO circuits (as measured by elevated PFHb) is an independent predictor of mortality.

## Materials and Methods

### Patient information and data collection

This is an observational study of consecutive patients who received ECMO support at Tampa General Hospital from 2007 till 2013. The Institutional Review Board (IRB) at the University of South Florida approved the study and waived the need for patient consent (IRB number Pro0000580).

A total of 154 patients received ECMO support during the study time. A retrospective chart review was performed with collection of clinical and laboratory data. These included variables comprising patients’ demographics, comorbidities, indications for ECMO support, ECMO characteristics, laboratory values during ECMO, transfusion requirements, outcomes and complications. Twenty-four-hour post-ECMO PFHb and lactate dehydrogenase (LDH) were collected and used as markers of hemolysis. PFHb was analyzed by derivative spectrometry (Molecular Devices, Sunnyvale, CA) as described in more detail elsewhere [16]. We used the cutoff value of PFHb> 50 mg/dL established by the extracorporeal life support organization (ELSO) as a marker of severe hemolysis [17].

### Study endpoints

The primary study outcome was to determine independent predictors of in-hospital mortality. Secondary outcomes included successful weaning from ECMO, bridging to other therapy and long-term survival. Successful weaning from ECMO was defined as survival for > 48 hours after ECMO removal [18]. Those who were weaned off ECMO support but died within 48 hours were defined as unsuccessfully weaned. Patients were followed after discharge to determine their survival days since ECMO explantation. Data of days survived for those discharged alive from the hospital was obtained through social security search and confirmed with phone interview.

### Clinical management

The ECMO pumps used during the study period was mainly Bio-Medicus BP80 (Medtronic, Inc. MN) or less commonly Thoratec Centrimag pump (Thoratec, Pleasanton, CA). Anticoagulation was achieved by intravenous infusion of heparin at a rate of 30–60 units/kg/hr to maintain an activated partial thromboplastin time (PTT) between 50–60 seconds. Plasma free hemoglobin and other coagulation parameters were frequently checked. Inotropic support was continued in cases of circulatory failure to facilitate left ventricular emptying and prevent over distension of the left ventricle. Diuretics were used to maintain negative fluid balance in subjects receiving ECMO for cardiac indications to decrease pulmonary vascular congestion. If the patient became hemodynamically stable with improvement in left ventricular contractility on echocardiography (in those with left ventricular dysfunction), inotropes were gradually weaned. The oxygen saturation was continuously monitored until the mixed venous oxygen saturation was more than 70%, and the pump flow was reduced gradually to 500 ml/min. ECMO support was withdrawn either with continued stability of the patient’s hemodynamic profile [19] or if its continuation was deemed futile. We considered the care futile in case of severe brain damage of absence of heart or lung recovery in those who are not a ventricular assist device or transplant candidate.

### Statistical analysis

Primary analysis compared survivors and non-survivors. Continuous variables were expressed as mean ± standard deviation and compared using Student t-test for normally distributed variables and Wilcoxon rank-sum test for non-normally distributed variables. Categorical variables were described as counts and percentages and were compared using the Chi-square or Fisher exact test when the expected frequency was less than 5. Univariate analysis was used to compare differences in various variables between survivors and nonsurvivors. In order to examine independent risk factors for mortality after ECMO support and avoid confounding effect, we used a Cox proportional hazard method. The variables used in the model were either those that achieved statistical significance at p < 0.01 upon univariate analysis or variables considered to be clinically relevant. For variables that remained significant after Cox proportional hazard analysis, cumulative survival curves were generated through the Kaplan-Meier approach for survivors and nonsurvivors and were compared using the log-rank test. A p value less than 0.05 was considered statistically significant. Data were analyzed using IBM SPSS 21.0 statistical software (IBM SPSS Version 21.0. Armonk, NY).

## Results

### Patient characteristics

A total of 154 patients received ECMO for either cardiac (n = 115) or pulmonary (n = 39) indications. Patients’ mean age was 51 years and 75.3% were males. Eighty-two percent of the subjects received venoarterial ECMO and 18% received venovenous ECMO. 48.7% of the cases were successfully weaned off ECMO. In-hospital mortality was 33% (51/154). Demographic, clinical and ECMO characteristics of both survivors and nonsurvivors are listed in table 1. Table 2 compares in-hospital survivors and nonsurvivors according to ECMO indication. Univariate analysis found no statistically significant difference between survivors and nonsurvivors according to patients’ disease that required ECMO support. Bridging to left ventricular assist device (LVAD), biventricular assist device (BIVAD), LVAD then cardiac transplant and bridging to transplant occurred with higher frequency among survivors compared with non-survivors (table 3).

**Table 1:** Demographics, clinical and extracorporeal membrane oxygenation characteristics among in-hospital survivors and nonsurvivors.

	All (n = 154)	Survivors (n = 51)	Nonsurvivors (n = 103)	p value
***Demographic and clinical characteristics***				
Age > 65 y % (n)	21.4% (33)	23.5% (12)	20.4% (21)	0.655
Male sex % (n)	75.3% (116)	74.5% (38)	75.7% (78)	0.869
BMI (mean ±SD)		28.1±7.6	29.7±7.8	0.237
Hypertension % (n)	62.3% (96)	66.7% (34)	60.2% (62)	0.435
Diabetes % (n)	36.4% (56)	41.2% (21)	34% (35)	0.382
Pre-ECMO ESRD % (n)	9.1% (14)	3.9% (2)	11.7% (12)	0.097
Prior cardiac surgery % (n)	35.1% (54)	31.4% (16)	36.9% (38)	0.499
PHTN % (n)	28.6% (44)	31.4% (16)	27.2% (28)	0.588
***Pre-ECMO characteristics***				
Pre-ECMO lactic acid (mean ±SD)		5±4.5	7.3±5.4	0.026
Pre- ECMO lactic acid > 4% (n)	39% (60)	27.5% (14)	44.7% (46)	0.05
Pre- ECMO lactic acid ≥ 10% (n)	21.4% (33)	11.8% (6)	26.2% (27)	0.053
AFib pre-ECMO% (n)	22.7% (35)	29.4% (15)	19.4% (20)	0.164
IABP pre-ECMO % (n)	11.7% (18)	7.8% (4)	13.6% (14)	0.296
***ECMO characteristics***				
24 h lactic acid (mean ±SD)		3.9±3.5	6±5.8	0.023
24 h LDH (mean ±SD)		477±232	2078±1687	0.14
PFHb 24 h (mean ±SD)		40 ± 17.5	119 ±173	0.029
PFHb 24 h > 50% (n)	11.7% (18)	3.9% (2)	15.5% (16)	0.002
Days on ECMO (mean ±SD)		4±3.5	6.4±7.6	0.01
ECMO duration ≥ 5 d % (n)	42.9% (66)	31.4% (16)	48.5% (50)	0.043
ECMO duration ≥ 7 d% (n)	25.3% (39)	13.7% (7)	31.1% (32)	0.02
***Blood products during ECMO***				
PRBC on ECMO (mean ±SD)		23±18	34.7±36.5	0.008
Plasma on ECMO (mean ±SD)		12.1±13	13.9±18.3	0.473
Platelet on ECMO (mean ±SD)		35±54.3	55.2±88.1	0.08
Cryoprecipitate on ECMO (mean ±SD)		27.5±31.6	25.7±38.6	0.775

ECMO: extracorporeal membrane oxygenation, BMI: body mass index, ESRD: end-stage renal disease, AFib: atrial fibrillation, MI: myocardial infarction, IABP: intra-aortic balloon pump, PHTN: pulmonary hypertension, LDH: lactate dehydrogenase, PFHb: plasma free Hb, PRBC: packed red blood cell, h: hour, d: day, n: number, SD: standard deviation

**Table 2:** Comparison between in-hospital survivors and nonsurvivors according to ECMO indication.

	All (n = 154)	Survivors (n = 51)	Nonsurvivors (n = 103)	p value
Cardiomyopathy % (n)	29.3% (43)	36.7% (18)	25.5% (25)	0.112
Cardiac arrest % (n)	10.2% (15)	6.1% (3)	12.2% (12)	0.195
Post MI cardiogenic shock % (n)	11.6% (17)	8.2% (4)	13.3% (13)	0.267
Cardiac surgery % (n)	12.2% (18)	12.2% (6)	12.2% (12)	0.613
Cardiac transplant % (n)	8.2% (12)	8.2% (4)	8.2% (8)	0.635
Lung transplant % (n)	10.2% (15)	14.3% (7)	8.2% (8)	0.191
Respiratory failure % (n)	10.9% (16)	10.2% (5)	11.2% (11)	0.547
Pulmonary embolism % (n)	4.1% (6)	2.1% (1)	5.1% (5)	0.356

MI: myocardial infarction

**Table 3:** Comparison of various bridging modalities from ECMO among in-hospital survivors and nonsurvivors.

	All (n = 154)	Survivors (n = 51)	Nonsurvivors (n = 103)	p value
Bridge to LVAD % (n)	10.4% (16)	21.6% (11)	4.9% (5)	0.001
Bridge to RVAD% (n)	0.6% (1)	2% (1)	0% (0)	0.331
Bridge to BIVAD% (n)	3.9% (6)	9.8% (5)	1% (1)	0.015
Bridge to LVAD then RVAD% (n)	2.6% (4)	3.9% (2)	1.9% (2)	0.404
Bridge to LVAD then transplant	2.6% (4)	7.8% (4)	0% (0)	0.011
Bridge to transplant % (n)	1.9% (3)	5.9% (3)	0% (0)	0.035

LVAD: left ventricular assist device, RVAD: right ventricular assist device, BIVAD: biventricular assist device.

### Univariate and multivariate analysis of risk factors for in-hospital Mortality

The results of univariate comparison of baseline demographic data, ECMO characteristics, ECMO indications and complications between nonsurvivors (died in-hospital) and survivors (weaned and discharged) are reported in tables 1, 2 and 4. Compared to nonsurvivors, survivors had lower pre-ECMO lactic acid level (p = 0.026), lower 24-hour lactic acid level (p = 0.023), shorter duration on ECMO (p = 0.01), fewer RBC transfusions on ECMO (p = 0.008) and lower value of PFHb 24-hours post ECMO implantation (p = 0.029). 24-hour PFHb> 50 mg/dL occurred in 3.9% versus 15.5% of survivors and nonsurvivors, respectively, p = 0.002. Plasma free hemoglobin > 50 mg/dL occurred in 16.7% versus 55% of survivors and nonsurvivors receiving venoarterial ECMO support, respectively, p = 0.032. With regards to venovenous ECMO, PFHb > 50 mg/dL occurred in 0% versus 80% of survivors and nonsurvivors, respectively, p = 0.04).

**Table 4:** Comparison of various complications encountered during extracorporeal membrane oxygenation among in-hospital survivors and nonsurvivors.

	All (n = 154)	Survivors (n = 51)	Nonsurvivors (n = 103)	p value
Acute renal failure % (n)	51.9% (80)	39.2% (20)	58.3% (60)	0.026
Bleeding % (n)	39.6% (61)	33.3% (17)	42.7% (44)	0.262
Arrhythmia % (n)	31.2% (48)	29.4% (15)	32% (33)	0.740
Hepatic dysfunction % (n)	41.6% (64)	25.5% (13)	49.5% (51)	0.004
Sepsis % (n)	15.6% (24)	13.7% (7)	16.5% (17)	0.637
Pericardial effusion % (n)	16.2% (25)	13.7% (7)	17.5% (18)	0.553
Pneumonia % (n)	12.3% (19)	7.8% (4)	14.6% (15)	0.233
Hemothorax% (n)	10.4% (16)	9.8% (5)	10.7% (11)	0.867
Hemorrhagic stroke % (n)	9.1% (14)	3.9% (2)	11.7% (12)	0.145
Mediastinitis% (n)	2.6% (4)	2% (1)	2.9% (3)	0.596
Ischemic stroke % (n)	8.4% (13)	5.9% (3)	9.7% (10)	0.319

**Fig 1:**
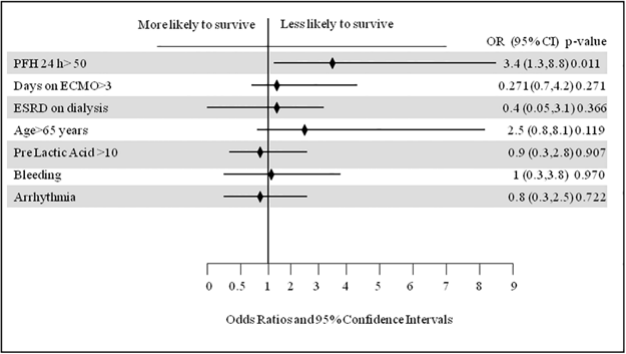
Cox proportional hazard method analysis of predictors of in-hospital mortality for patients receiving extracorporeal membrane oxygenation at Tampa General Hospital from 2007–2013.

**Fig 2:**
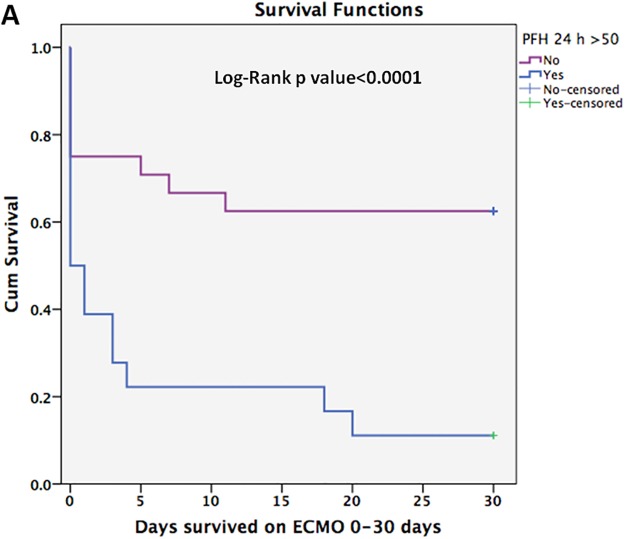
Kaplan-Meier cumulative survival curve comparing subjects with plasma free Hb>50 mg/dL or<50 mg/dL checked 24-hour post ECMO initiation for a follow up period of 30 days.

**Fig 3:**
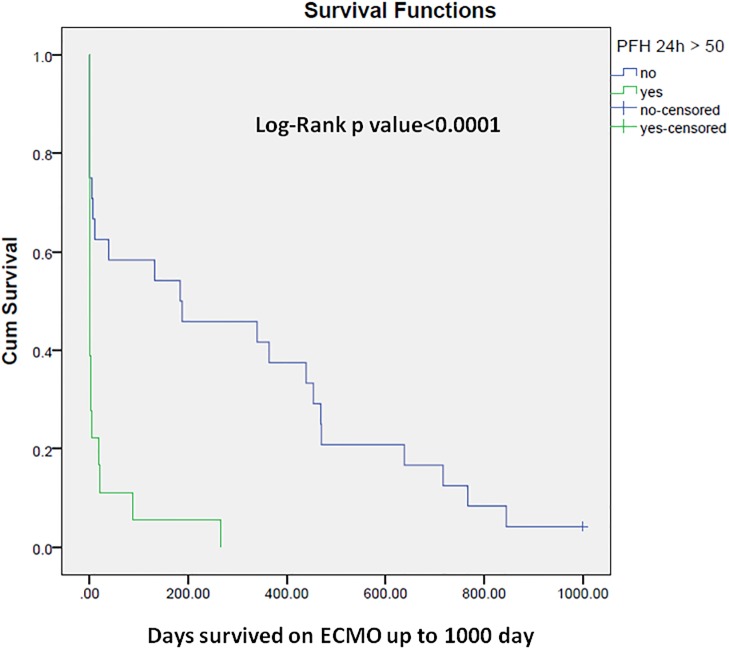
Kaplan-Meier cumulative survival curve comparing subjects with plasma free Hb>50 mg/dL or<50 mg/dL checked 24-hour post ECMO initiation up to 3 years of follow up.

Cox proportional hazard analysis identified PFHb> 50 mg/dL 24-hours post ECMO initiation as an independent predictor of mortality (OR = 3.4, 95% confidence interval [CI]: 1.3–8.8, p = 0.011). Fig 1 illustrates the results of Cox proportional hazard analysis of predictors of in-hospital mortality for patients receiving ECMO support. Figs 2 and 3 show the Kaplan-Meier curves comparing survival in patients with 24-hour post-ECMO PFHb levels > 50 mg/dL or < 50 mg/dL according to the follow up period.

### Complications

Table 4 lists the comparison of various complications encountered during ECMO support between survivors and nonsurvivors. The most common complications experienced in patients receiving ECMO support was acute renal failure (80/154, 51.9%) followed by hepatic dysfunction in (64/154, 41.6%) followed by bleeding (61/154, 39.6%). Nonsurvivors experienced higher incidence of acute renal failure (p = 0.026) and hepatic dysfunction (p = 0.004). There was a trend towards higher risk of hemorrhagic stroke in nonsurvivors (3.9% versus 11.7% in survivors and nonsurvivors respectively, p = 0.145). Plasma free hemoglobin did not predict the occurrence of non-fatal ECMO complications and levels were comparable among cases complicated with acute renal failure (p = 0.136), bleeding (p = 0.554), arrhythmia (p = 0.663), hepatic dysfunction (p = 0.885), sepsis (p = 0.891), pericardial effusion (p = 0.496), hemothorax (p = 0.677) and ischemic stroke (p = 0.892).

## Discussion

The current study found that PFHb> 50 mg/dL within the first 24 hours post-ECMO initiation was an independent mortality predictor. On the basis of this finding we have implemented a routine check of PFHb with awareness that PFHb levels >50 mg/dL is a sign of severe hemolysis (acceptable values of PFHb are 5–10 mg/dL) warranting investigation of the underlying etiology and correcting it, including the possibility of changing the pump head.

After destruction of RBCs, PFHb is cleared by the hemoglobin (Hb) scavengers. When the severity of hemolysis is beyond the capacity of the intravascular Hb-scavenging mechanisms, hemoglobinemia and hemoglobinuria occur in addition to Hb-mediated nitric oxide (NO) scavenging where Hb binds NO from the endothelium [20]. The subsequent depletion of NO (a potent vasodilator) can lead to increased systemic and pulmonary vascular resistance, increased thrombin formation, fibrin deposition, platelet aggregation, organ dysfunction and increased mortality rate [14]. Plasma free hemoglobin > 10 mg/dL was found to inhibit NO-induced vasodilation in vivo [21]. Moreover, the released iron (Fe) from hemolysis can result in Fe overload in 20% of the cases [22] which can lead to renal failure and increased lung permeability [23]. In addition, the resulting hyperbilirubinemia after cardiac bypass surgery has been associated with increased mortality [24]. Sublethal RBC damage leading to decreased deformability and increased aggregability will lead to its removal by the spleen, lower oxygen content and decrease ability to enter small capillaries thereby interfering with tissue oxygenation [25] and leading to end organ dysfunction [20]. Taking all this into account, one should be more concerned about the adverse effect of hemolysis rather than the developing hemolysis-induced anemia.

Pump technology is a main determinant of hemolysis in patients receiving ECMO support. It was found that centrifugal pumps (CF) when compared with roller pump based systems exhibit superior blood handling capabilities [26] and therefore are more accepted. Centrifugal pumps however may cause microcavitation and hemolysis. There is also a variable degree of hemolysis according to type of CF pump used. First generation CF pumps are larger, produce areas of stasis and friction points which adversely affected blood components [27]. A recent study by Bottrell and colleagues evaluated the changes in PFHb with three different pumps and found that the Levitronix PediVAS was associated with significantly less hemolysis than the Rotaflow (p<0.05) and the DP3 (p<0.05) [27]. Lawson and colleagues compared three CF pumps with a roller pump in the incidence of hemolysis by measuring the PFHb and found that 2 centrifugal pumps (the Cobe Revolution and the Jostra Rotaflow) compared favorably with the Cobe Century roller occlusion blood pump regarding the amount of hemolysis produced [28]. The BioMedicus BP80 (Medtronic, Inc. MN) was the main pump used during our study period. The Thoratec Centrimag pump (Thoratec, Pleasanton, CA) was infrequently used due to the significantly higher cost. The later pump characteristically requires lower RPM compared with the former. It is expected that with the continued advancement of CF pump technology, newer pump designs will reduce the severity of hemolysis.

Pre-ECMO mortality predictors are obviously more efficient and useful than predictors during ECMO support as they help in deciding which patients should and which should not get ECMO. However, identifying predictors of mortality during ECMO support is also useful as they help in prognostication and guide management. Among these factors that we have suspected from our experience is hemolysis, which is usually related to pump malfunction. This has been more studied with ventricular assist device where hemolysis was found to be an independent mortality predictor (as it usually reflects pump thrombosis) which requires pump exchange or cardiac transplantation to avoid death of the patient. The awareness of PFHb > 50 as an independent mortality predictor during ECMO will prompt its routine check as well as immediate circuit examination.

The current study is subject to all limitations inherent to non-randomized studies. The design is a retrospective one and the number of cases is moderate. Herein, we aim to emphasize the value of PFHb, a marker of hemolysis, as an independent mortality predictor in patients receiving ECMO support because it is a proxy for suboptimal ECMO mechanics. We recommend that physicians check PFHb level 24-hour post-ECMO initiation to assess their short-term prognosis. We speculate that daily checking of PFHb (after first 24 hours) might be of value and this needs to be tested in future studies. Further technical advances should be made to develop less traumatic ECMO devices with lower risk of hemolysis. Our study also showed a favorable incidence of in-hospital mortality compared with other studies and a relatively good long-term outcome of survivors. The main predictor of outcome remains the severity of the primary pathology. Initiating ECMO support has to be considered on the basis of global risk profile evaluation of each patient, taking into account the pre-procedural chances of success, rather than individual risk factor assessment.
